# Clinical Challenges to Current Molecularly Targeted Therapies in Lung Cancer

**DOI:** 10.21767/2254-6081.100030

**Published:** 2015

**Authors:** Gagan Chhabra, Ashley Eggert, Neelu Puri

**Affiliations:** Department of Biomedical Sciences, University of Illinois College of Medicine, Rockford, Illinois, USA

**Keywords:** NSCLC, Molecularly targeted therapies, TKI, Resistance

## Abstract

Lung cancer is difficult to treat with a poor prognosis and a five year survival of 15%. Current molecularly targeted therapies are initially effective in non-small cell lung cancer (NSCLC) patients; however, they are plagued with difficulties including induced resistance and small therapeutically responsive populations. This mini review describes the mechanism of resistance to several molecularly targeted therapies which are currently being used to treat NSCLC. The major targets discussed are c-Met, EGFR, HER2, ALK, VEGFR, and BRAF. The first generation tyrosine kinase inhibitors (TKIs) resulted in resistance; however, second and third generation TKIs are being developed, which are generally more efficacious and have potential to treat NSCLC patients with resistance to first generation TKIs. Combination therapies could also be effective in preventing TKI resistance in NSCLC patients.

## Introduction

The focus of current lung cancer treatment has been shifted from more traditional options to newly developed molecularly targeted therapies. Many of the molecularly targeted therapies are utilized to target specific biomarkers that are commonly overexpressed and have important roles in tumorigenesis; these biomarkers contribute to cancer-related processes such as cell proliferation, survival and migration. While initially effective, many targeted therapies have been associated with increased drug resistance after their initial use. Acquired resistance to current molecularly targeted therapies in lung cancer presents a major clinical challenge. Current research focuses on identifying potential novel biomarkers and mechanisms involved in resistance to these therapies. There are several clinical challenges associated with current molecularly targeted therapies including the induction of various types of resistance mechanisms, which are not clearly defined, and the lack of effective combinatorial therapies designed to prevent and overcome the problem of drug resistance in lung cancer.

## Current Therapies

Common molecularly targeted therapies target receptor tyrosine kinases (RTKs) including hepatocyte growth factor receptor (HGFR/c-Met), epidermal growth factor receptor (EGFR), human epidermal growth factor receptor 2 (HER2), anaplastic lymphoma kinase (ALK), and endothelial growth factor receptor (VEGFR), which are commonly mutated in NSCLC cases [1]. Recently, v-Raf murine sarcoma viral oncogene homolog B1 (BRAF) has also been shown as a potential target for treatment of advanced NSCLC patients having mutated BRAF. Mutations in these RTKs cause uncontrolled upregulation and amplification of various downstream signaling pathways including MAP kinase (mitogen-activated protein kinases), PI3K (phosphoinositide 3-kinase)/AKT (protein kinase B) and mTOR (mammalian target of rapamycin) pathways; these pathways are responsible for cell survival, proliferation, migration, protein synthesis, and angiogenesis of cancerous cells [2]. In order to inhibit cell growth and proliferation, many tyrosine kinase inhibitor inhibitors (TKIs) have been developed that act by binding to RTKs and inhibiting their downstream signaling cascades [1].

c-Met is a RTK for the ligand hepatocyte growth factor (HGF), which is secreted by mesenchymal cells and cancer cells [3]. There have been several monoclonal antibodies designed to target c-Met/HGF including rilotumumab (AMG 102), ficlatuzumab (AV- 299), onartuzumab (MetMAb), as well as TKIs including tivantinib (ARQ-197), cabozantinib (XL184/BMS-907351), crizotinib (PF- 2341066), and foretinib (XL880, GSK1363089) [4]. For each of these TKIs, resistance is a major concern [5] and several mechanisms for resistance have already been proposed. One study showed that MET-dependent NSCLC cells that had become resistant displayed high levels of c-Met and KRAS (kirsten rat sarcoma viral oncogene homolog) amplification, leading to downstream MAP kinase activity [6]. Another study found that inhibition of c-Met in Met-amplified NSCLC led to activation of the EGFR pathway [7]. However, in a gastric carcinoma cell-line, a mutation in the c-Met activation loop has been shown to destabilize autoinhibitory conformational change, ultimately causing constitutive expression which could be a possible mechanism of c-Met TKI resistance [8].

Epidermal growth factor receptor (EGFR) is a transmembrane receptor that plays an essential role in regulating cell proliferation, survival, and growth [9]. EGFR TKIs inhibit receptor phosphorylation and downstream signaling by binding to the intracellular EGFR TK domain. The first generation of EGFR TKIs bind reversibly to the ATP binding site of the EGFR TK domain; due to high binding affinity for this domain, an inhibition of RTK activity is observed [10]. However, prolonged use of EGFR-TKIs can lead to distinct drug resistance patterns. The dominant resistance pattern is a common T790M secondary mutation. The T790M mutation induces resistance by interfering with TKIs binding to the ATP binding domain [11]. D761Y, T854A and L747S (Table 1) are additional secondary mutations that cause resistance; these arise subsequent to the EGFR TKI sensitizing L858R mutation [12]. Our recent studies indicate that the activation of alternative signaling pathways such as PI3K/mTOR and Wnt may also cause resistance to EGFR TKIs [13,14]. A second generation EGFR TKI, afatinib, which irreversibly binds to the ATP binding pocket of EGFR, was suggested to have potential to overcome TKI resistance. This inhibitor is efficacious in NSCLC patients who have T790M mutation which confers resistance to EGFR TKIs such as erlotinib [15], however it also has been shown to inhibit wild type EGFR that may result in dose limiting toxicities. AZD9291, CO-1686, and HM61713 are the third generation of TKIs that target both the sensitizing mutations and the T790M resistance mutation while sparing the wild type EGFR [16,17] and show potential to overcome resistance. HER2, another member of the EGFR family, also activates downstream signaling pathways such as RAS, PI3K, MAPK, and SRC. The HER2 TKI lapatinib and the HER2 antibody trastuzumab are originally very effective at blocking HER2 signaling, but their effectiveness decreases over time. This may be due to the T798M mutation [18]; however, the mechanism through which the T798M mutation confers resistance may be due to increased EGFR ligand production [18].

The anaplastic lymphoma kinase (ALK) is a RTK typically expressed in the central and peripheral nervous system regions [19,20]. ALK gene amplification, mutation and rearrangement are known to be associated with tumor development in lung cancer patients [21,22]; approximately 5% of NSCLC cases are diagnosed with ALK gene rearrangement [22]. Crizotinib, a small-molecule ALK TKI was the first FDA approved drug to treat patients with ALK-rearranged NSCLC. However, the efficacy of crizotinib is limited to approximately one year due to the emergence of resistance patterns. Point mutations including L1196M, C1156Y, G1269A and F1174L in the kinase domain of ALK have been observed in biopsies from patients treated with crizotinib, a first generation ALK TKI, and have been found perturbing crizotinib binding to render it less effective [23–25]. Another study identified G1202R, S1206Y and 1151Tins point mutations in crizotinib treated ALK-positive NSCLC patients. Ceritinib, alectinib, and AP26113 are amongst the second generation of ALK TKIs with improved selectivity and potency compared to crizotinib. However, mutations in the ALK gene conferring resistance to alectinib (G1123S, G1202R, I1171T/N/S, and V1180L) and ceritinib (G1202R and F1174C/V) have also been found (Table 1) [26–31].

Overexpression of vascular endothelial growth factor (VEGF), an angiogenic factor, and its receptors are related to poor prognosis in NSCLC patients [32]. Bevacizumab (a monoclonal antibody that targets VEGF) and aflibercept (a recombinant fusion protein that binds strongly to VEGF) are being explored clinically to block VEGF pathways in NSCLC patients [33,34]. Acquired resistance to anti-VEGF therapy usually occurs via several distinct mechanisms [35] including expression of additional proangiogenic pathways including platelet derived growth factor (PDGF) and fibroblast derived growth factor (FGF) [36].

BRAF (v-Raf murine sarcoma viral oncogene homolog B1) is a member of the RAF serine/threonine protein kinases family. Mutations in BRAF have been shown to be associated with tumor development in NSCLC with a frequency of 2–3%. Recently, a BRAF inhibitor dabrafenib, the first drug of its class, is shown to be effective for the treatment of advanced NSCLC patients with BRAF V600E mutation in a phase II clinical study [37]. However, one study reported acquired resistance to dabrafenib in a patient after 8 months of response. An acquired G12D mutation (Table 1) in KRAS has been suggested to be primarily responsible for acquired dabrafenib resistance in this patient [38]. Further studies are required to understand the therapeutic potential of this inhibitor.

## Conclusion

Although current molecularly targeted therapies are very effective for NSCLC patients, almost all patients eventually acquire resistance to these therapies. To combat this resistance against first generation TKIs, second and third generation TKIs have been developed. These new generations of TKIs are either completing clinical trials or have been FDA approved to treat NSCLC patients. However, their therapeutic potential needs to be further validated and established. Various secondary mutations and alternative signaling pathways have been identified as distinct resistance patterns for several TKIs targeting EGFR, c-Met, and ALK. However, further studies are required to determine the specific mechanisms of acquired resistance to HER2, VEGFR and BRAF. Combinatorial strategies could be effective in overcoming TKI resistance in lung cancer patients. These strategies may require targeting both mutations involved in resistance and alternative signaling pathways.

## Figures and Tables

**Table 1:** Mutations conferring sensitivity or resistance to tyrosine kinase inhibitors (TKIs) therapies.

**EGFR mutations**	
L858R*	Leucine to arginine at position 858
T790M	Threonine to methionine at position 790
D761Y	Aspartic acid to tyrosine at position 761
T854A	Threonine to alanine at position 854
L747S	Leucine to serine at position 747
**HER2 mutation**	
T798M	Threonine to methionine at position 798
**ALK mutations**	
L1196M	Leucine to Methionine at position 1196
C1156Y	Cysteine to tyrosine mutation at position 1156
G1269A	Glycine to alanine at position 1269
F1174L	Phenylalanine to leucine at position 1174
G1202R	Glycine to arginine at position 1202
S1206Y	Serine to tyrosine at position 1206
1151Tins	Threonine insertion at amino acid 1151
G1123S	Glycine to Serine at position 1123
I1171T/N/S	Isoleucine to threonine/asparagine/serine at position 1171
V1180L	Valine to leucine at position 1180
F1174C/V	Phenylalanine to cysteine/valine at position 1174
**BRAF mutation**	
V600E	Valine to glutamic acid at position 600
**KRAS mutation**	
G12D	Glycine to aspartic acid at position 12

* Mutations which confer sensitivity to TKIs.
